# Practical implementation of generative artificial intelligence systems in healthcare: A United States perspective

**DOI:** 10.1016/j.fhj.2024.100166

**Published:** 2024-09-19

**Authors:** Barclay Burns, Bo Nemelka, Anmol Arora

**Affiliations:** a Utah Valley University, UT, United States; b Cambridge Judge Business School, Cambridge, United Kingdom; c Sanford Health, SD, United States; d University of Cambridge, Cambridge, United Kingdom

## Introduction

**Fig. 1:**
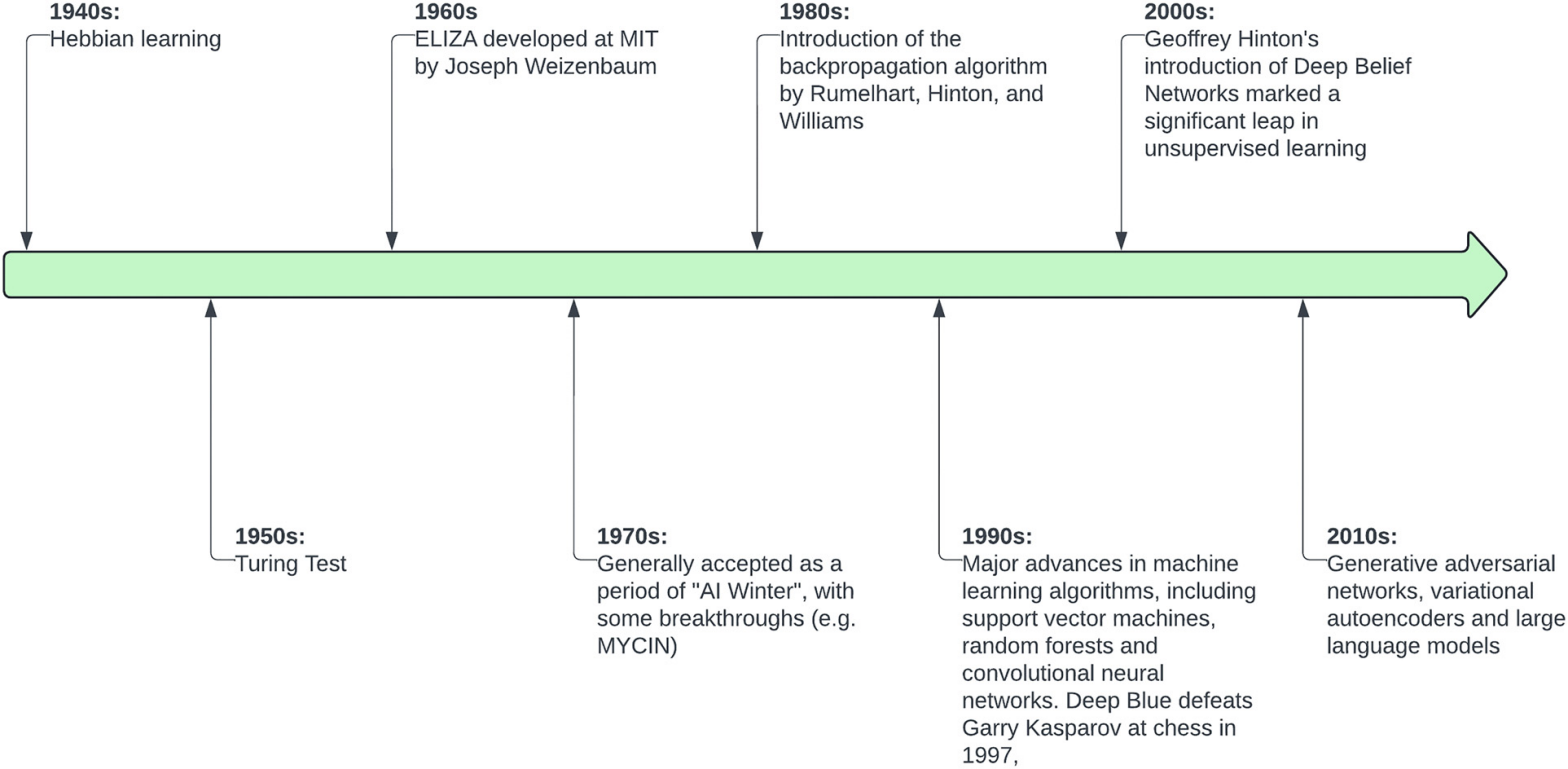
Key breakthroughs in the field of generative artificial intelligence since the 1940s (note that this is not a comprehensive history).

Generative artificial intelligence (AI) represents a subset of AI that focuses on generating new content after being trained on existing data, in contrast to deductive AI systems, which seek to analyse data to derive conclusions or predictions. The new content produced by generative AI technologies can include text, images, audio or simulations. Generative AI algorithms have evolved significantly since their inception. ‘Large language models’ (LLMs) are a subset of generative AI models and, while the terms are sometimes (inappropriately) used synonymously, the field of generative AI is much broader. The evolution of AI systems has included key breakthroughs over the past century (Fig. 1), including Hopfield networks in the 1980s, Boltzmann machines in the 1990s and the introduction of deep belief networks in the 2000s. A transformative paper was published in 2014 in which Ian Goodfellow proposed generative adversarial networks (GANs), enabling the generation of highly realistic images and data.^1^ This was closely followed by the introduction of the modern transformer model by Google in 2017, which revolutionised natural language processing.^2^ The first generative pre-trained transformer (GPT) system was produced by OpenAI in 2018, with companies and research groups subsequently developing numerous LLMs, some of which are easily accessible to the public through application programming interfaces (APIs). Interestingly, there has been a recent trend in AI breakthroughs being attributable to private companies rather than solely academic environments. While academia remains vital for foundational research, private companies have arguably become the primary drivers of AI advancement, perhaps fuelled by their ability to invest heavily in the necessary resources and infrastructure.

## Applications of generative AI in healthcare

LLMs are machine learning models programmed to predict text sequences, generally having been trained on large swathes of data from the internet and text corpora.^3^ By producing high-fidelity output, these models can generate human-like text, including in the form of chatbot responses or radiology reports. Innovation into LLMs has largely been driven by large technological companies, such as OpenAI and Meta, the latter of which has published their model ‘Llama 3’ open source.^4^ One of the most challenging precepts of digital health implementation is the need to integrate with electronic health records, which is why the announced partnership between Epic and Microsoft to integrate GPT-4 in Epic was a significant breakthrough.^5^

There is ongoing work to attempt to automate patient note summarisation, radiology reporting and discharge summary preparation using LLMs. However, there are limitations due to the models’ tendency to generate outputs based on the most probable sequences of words, which can lead to critical errors when dealing with small but significant negative words like ‘no’. Radiology reports and patient summaries require precise and unambiguous language, as the presence or absence of specific terms (eg ‘no fracture’ vs ‘fracture’) can drastically alter the clinical interpretation. LLMs, despite their sophisticated contextual understanding, are prone to occasional inaccuracies, hallucinations and misinterpretations, particularly with negations, which are critical in medical contexts. While the ideal solution would be to re-engineer LLMs to prevent hallucinations being generated, emerging solutions are more targeted towards fact-checking the output of a model before presenting it to the user. For example, by measuring uncertainty metrics (eg token probability or entropy) of the LLM that can be used as a proxy for a confidence score of the output, with low scores more likely to represent hallucinations.

As well as medical text, generative algorithms can be used to synthesise other forms of health data. Synthetic data are conventionally defined as high-fidelity data generated by machine learning algorithms, designed to mimic real data.^6^ Synthetic data can be created in a range of forms, including images, videos or tabular datasets. In population health analytics, synthetic data present a novel opportunity to produce large datasets that can be shared for analysis while preserving privacy. These synthetic data can be used to model disease outbreaks, predict health trends and plan public health interventions without compromising individual patient confidentiality. It has been suggested that synthetic data could be used for the early training of risk prediction models, which typically require access to large national datasets to accelerate and de-risk the development of predictive models.^7^ Aside from synthetic data, generative AI also enhances risk prediction modelling through its ability to simulate clinical scenarios. By leveraging LLMs and other generative algorithms, AI systems can create realistic datasets that capture a wide range of clinical variations and outcomes. These simulations enable the testing and validation of predictive models under various hypothetical conditions, increasing the likelihood that the models are robust and adaptable to real-world complexities. The NHS has already led a pilot with the use of synthetic data for A&E services.^8^ There is also a national Simulacrum with national-level synthetic cancer repository data from the UK.^9^ In 2024, the state of Utah became the first legislative body to create a specific legal provision for synthetic data, defining it as a type of anonymised data.^10^

## Factors affecting successful implementation

Innovation is a process broadly characterised by three stages: invention, development and implementation.^11^ Notably, while Fig. 1 presents a series of laudable AI inventions, a figure demonstrating successful real-world AI implementations would be much narrower. Healthcare as an industry is recognised as one of the fastest moving for new inventions, in part due to the moral imperative of governments and society to improve patient care. However, the actual adoption of digital innovations tends to lag behind wider society.^12^ This is particularly notable in publicly funded hospitals, where expenditure on digital infrastructure may be viewed less favourably by governments than other investments that improve patient care more immediately in the eyes of the public, such as improved staffing levels or improving physical equipment.

### Human-centred design

Any digital health innovation must be acceptable to the public, who are the end customers even in a health system in which they are not the direct payer. Successful integration of generative AI in healthcare requires active involvement from a diverse group of stakeholders, including patients, healthcare providers, academics, engineers and health systems administrators. Collaborative efforts ensure that the technology addresses real-world needs and incorporates multidisciplinary perspectives.

Human-centred design prioritises the needs and experiences of end users, which, in healthcare, include both patients and healthcare providers. As well as these stakeholders, it is advisable to engage with data science and cybersecurity teams within hospitals, from the early stages of generative AI implementation as they oversee the security, reliability and ethical use of new technological systems. Early collaboration facilitates the development of robust, secure and compliant AI solutions that integrate seamlessly with existing healthcare infrastructures. For example, ambient clinical AI systems that use generative AI to automatically transcribe consultations and create clinical documentation, were born out of the recognition that doctors can spend up to 2 hours on documentation and admin work for each hour spent directly facing patients. These systems are being piloted in the UK with TORTUS partnering with Great Ormond Street Hospital and in the US with Intermountain Health deploying DAX Copilot across its enterprise.^13^^,^^14^ Ethical challenges of ambient AI systems include the handling of large amounts of continuously collected sensitive data and empirical evidence from real-world deployment for its acceptability to patients and providers is eagerly awaited.^15^

### Principal–agent relationship

The principal–agent problem is a fundamental organisational theory that arises when one party (principal) delegates work and some decision-making authority to another party (agent).^16^ The implementation of generative AI in healthcare introduces a novel principal–agent dynamic. In the context of AI, clinicians (principals) delegate decision-making authority to AI systems (agents). The principal–agent problem dictates that, in most cases, there will be divergence between the interests of the principal and the agent, such that the behaviours taken by the agent do not maximise the utility for the principal. The principal–agent problem is not a new phenomenon in healthcare, which already involves numerous stakeholders who work together, sometimes with divergent interests:

- patients
- doctors
- administrators
- nurses
- parents (in paediatric populations).

Currently within healthcare, the most commonly cited principal–agent problem is provider-induced demand, whereby health providers (agents) may make decisions about treatments that align with their own self-interests rather than that of the patient (principal) in private healthcare models.^17^ A shift to further complicate these dynamics with the addition of AI necessitates a re-evaluation of trust, accountability and oversight mechanisms to ensure that AI-driven decisions align with ethical standards and patient wellbeing.

### Sustainable and publicly acceptable business model

In the United States, health innovation tends to be driven by for-profit enterprises in a competitive oligopoly market. Although market forces typically align with the objective of improving patient outcomes, the fiscal responsibility to deliver timely return on investment to shareholders may not always align with the moral imperative to improve patient care. Therefore, certain health services require a different vehicle for enterprise, such as in the form of state support, B corporations or in the healthcare utility model. State support is the most common mechanism and a simplified high-level overview of available pathways is illustrated in Fig. 3. The health utility model is a particularly novel form of care delivery as a non-profit initiative with a social mission to treat health services as a commodity and provide them at the lowest possible cost. Rather than sourcing funding from private investors, banks or venture capital firms which seek a profitable return on investment, health utility models rely upon the users of the utility to provide funding commitments, with the mutual understanding that those users will ultimately benefit from the investment in the long term.

### Ethically sourced and robust training data

The quality and ethics of training data are paramount to the success of generative AI in healthcare. Datasets must be representative, unbiased and sourced ethically to ensure the AI systems perform accurately and fairly across diverse patient populations. Medical research suffers from health data poverty, whereby certain demographic groups tend to be underrepresented in health datasets that are being used to train AI models.^18^ As a result, the AI models may exhibit algorithmic bias when applied to these groups. There are emerging efforts to develop standards for the curation of datasets to ensure that generative AI models are generalisable and applicable to the general population.^19^

### Post-market surveillance and safety monitoring

Post-market surveillance and safety monitoring are critical components of generative AI implementation in healthcare. Continuous monitoring of AI systems' performance in real-world settings helps identify and mitigate risks, ensuring ongoing safety and efficacy. Regulatory bodies and healthcare organisations must establish robust frameworks for tracking and responding to adverse events, system failures, and unexpected outcomes. The American Medical Association have repeatedly call for the appropriate governance policies and have advised that physicians should only engage with generative AI systems when they have been provided with adequate about the products and their risks.^20^

## Future considerations

The practical implementation of generative AI systems in healthcare promises to enhance patient care, streamline operations, and act as a platform for further innovation. However, realising this potential requires careful consideration of ethical, regulatory and operational challenges. Ensuring the safe implementation of generative AI in healthcare is a shared responsibility between government and healthcare organisations. In the United States, 40 payers and providers have collaborated with the White House to develop and pledge to the ‘Healthcare AI Commitments Initiative’ that provides a harmonised framework towards AI innovation. These voluntary commitments represent an important step to align industry action with principles that ensure AI implementation is fair, appropriate, valid, effective and safe (FAVES), which will likely lay groundwork towards further regulation in coming years.^21^^,^^22^

**Fig. 2:**
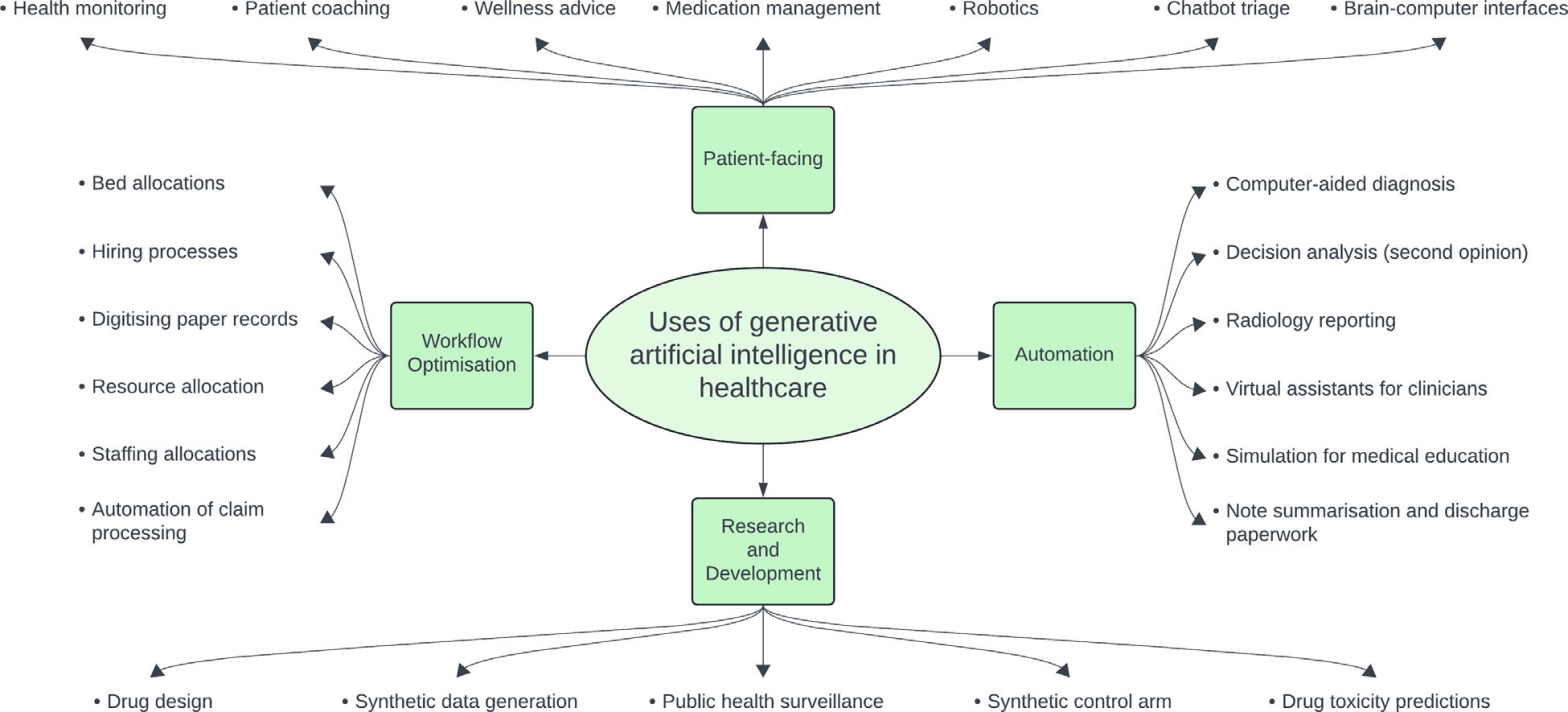
Diagrammatic illustration of use cases of generative artificial intelligence in healthcare.

Only by involving diverse stakeholders, ensuring robust data practices and maintaining vigilant post-market surveillance can healthcare sector harness the transformative power of generative AI while also safeguarding patient welfare and public trust, (Fig. 2).

**Fig. 3:**
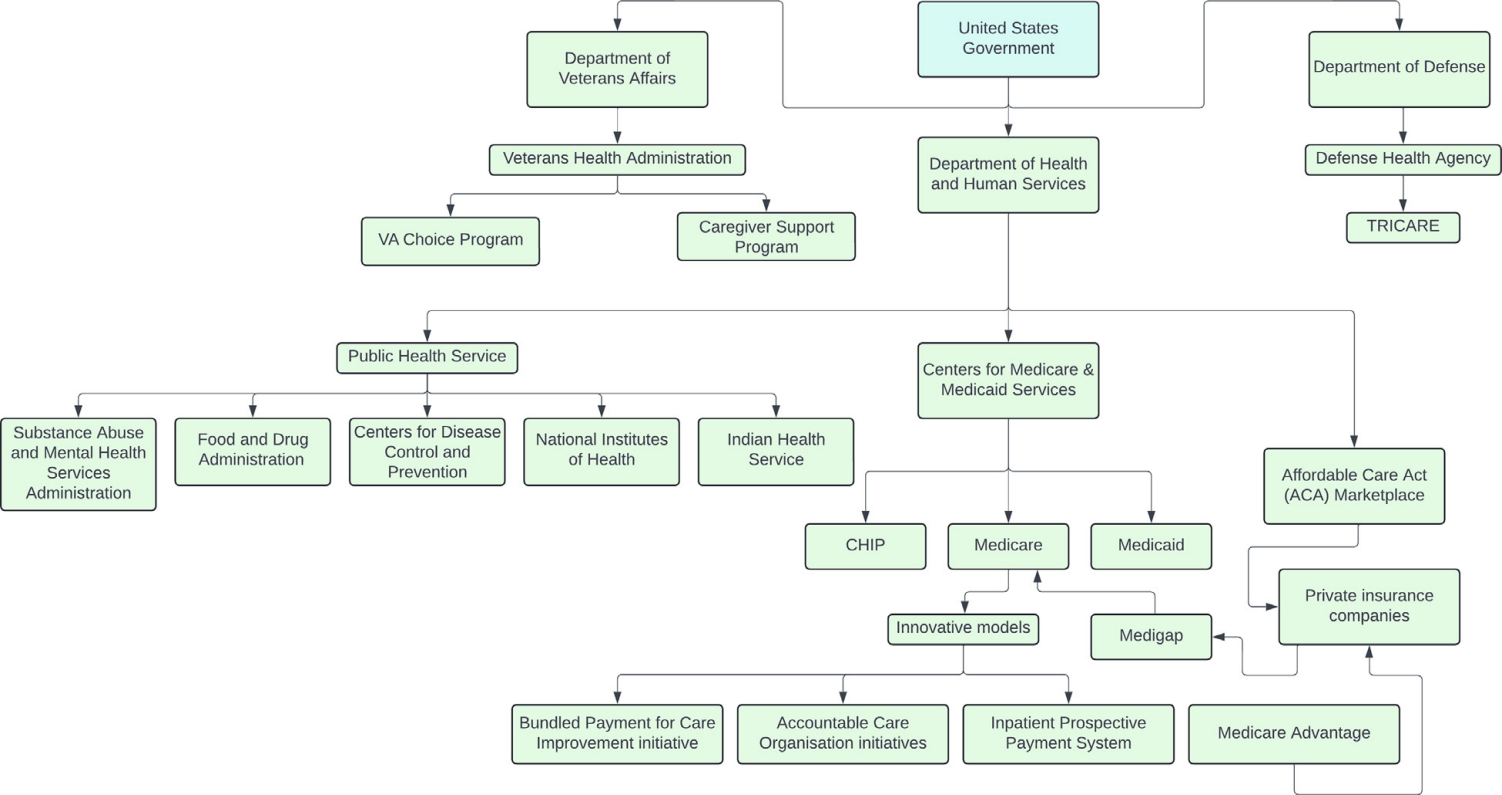
A simplified high-level overview of governmental funding programs available for healthcare services in the United States.

## CRediT authorship contribution statement

**Barclay Burns:** Conceptualization, Writing – review & editing. **Bo Nemelka:** Conceptualization, Writing – review & editing. **Anmol Arora:** Conceptualization, Writing – review & editing.
